# 3-Deazaneplanocin A (DZNep), an Inhibitor of *S*-Adenosylmethionine-dependent Methyltransferase, Promotes Erythroid Differentiation*[Fn FN2]

**DOI:** 10.1074/jbc.M114.548651

**Published:** 2014-02-03

**Authors:** Tohru Fujiwara, Haruka Saitoh, Ai Inoue, Masahiro Kobayashi, Yoko Okitsu, Yuna Katsuoka, Noriko Fukuhara, Yasushi Onishi, Kenichi Ishizawa, Ryo Ichinohasama, Hideo Harigae

**Affiliations:** From the Departments of ‡ Hematology and Rheumatology,; § Molecular Hematology/Oncology, and; ‖ Hematopathology, Tohoku University Graduate School, Sendai 980-8575, Japan and; the ¶ Clinical Research, Innovation and Education Center, Tohoku University Hospital, Sendai 980-8575, Japan

**Keywords:** Erythropoeisis, Gene Transcription, Hemoglobin, Histone Deacetylase, Histone Methylation, DZNep, ETO2, EZH2

## Abstract

EZH2, a core component of polycomb repressive complex 2 (PRC2), plays a role in transcriptional repression through histone H3 Lys-27 trimethylation and is involved in various biological processes, including hematopoiesis. It is well known that 3-deazaneplanocin A (DZNep), an inhibitor of *S*-adenosylmethionine-dependent methyltransferase that targets the degradation of EZH2, preferentially induces apoptosis in various hematological malignancies, suggesting that EZH2 may be a new target for epigenetic treatment. Because PRC2 participates in epigenetic silencing of a subset of GATA-1 target genes during erythroid differentiation, inhibition of EZH2 may influence erythropoiesis. To explore this possibility, we evaluated the impact of DZNep on erythropoiesis. DZNep treatment significantly induced erythroid differentiation of K562 cells, as assessed by benzidine staining and quantitative RT-PCR analysis for representative erythroid-related genes, including globins. When we evaluated the effects of DZNep in human primary erythroblasts derived from cord blood CD34-positive cells, the treatment significantly induced erythroid-related genes, as observed in K562 cells, suggesting that DZNep induces erythroid differentiation. Unexpectedly, siRNA-mediated EZH2 knockdown had no significant effect on the expression of erythroid-related genes. Transcriptional profiling of DZNep-treated K562 cells revealed marked up-regulation of *SLC4A1* and *EPB42*, previously reported as representative targets of the transcriptional corepressor ETO2. In addition, DZNep treatment reduced the protein level of ETO2. These data suggest that erythroid differentiation by DZNep may not be directly related to EZH2 inhibition but may be partly associated with reduced protein level of hematopoietic corepressor ETO2. These data provide a better understanding of the mechanism of action of DZNep, which may be exploited for therapeutic applications for hematological diseases, including anemia.

## Teaser

**Background:** *S*-adenosylmethionine-dependent methyltransferase inhibitor, DZNep, targets the degradation of histone methyltransferase EZH2 that catalyzes H3K27 trimethylation.

**Results:** DZNep induced erythroid-related genes, which may not be directly related to EZH2 inhibition but may be partly associated with reduced protein level of hematopoietic corepressor ETO2.

**Conclusion:** DZNep has the capacity to induce erythroid differentiation.

**Significance:** Our data may be exploited for therapeutic applications for hematological diseases, including anemia.

## Introduction

The differentiation of hematopoietic stem cells into specific progenitor cells and ultimately into diverse blood cell types is controlled by various transcription factors, represented by the GATA factors (1). In the context of erythropoiesis, GATA-1 activates or represses the expression of various genes by recognizing the DNA binding consensus sequence (A/T)GATA(A/G) through dual zinc finger motifs, which are characteristic of the GATA family (2). GATA-1 forms a complex with another master regulator of hematopoiesis, the basic-helix-loop-helix transcription factor SCL/TAL1 (1, 3, 4). SCL/TAL1 is a master regulator of hematopoiesis that binds E-boxes and the non-DNA-binding components of LMO2 and LDB1 (1, 3–10), which contributes to the activation of GATA-1 target genes.

Although it has been known that a subset of genes could be directly repressed by GATA-1, the partners for GATA-1 in gene repression are less clear (11). Hematopoietic corepressor ETO2 associates with the GATA-1·SCL/TAL1 complex and confers transcriptional repression mediated through interaction with histone deacetylases (HDACs)^2^ (12–16). GATA-1 is also thought to facilitate gene repression via association with the NuRD (nucleosome remodeling and deacetylation) complex, which may be mediated by FOG-1 (friend of GATA-1) (17, 18), as well as via the transcriptional corepressor Gfi-1 (19). GATA-1 can recruit polycomb repressive complex 2 (PRC2), a multiunit complex comprising the EZH2 histone methyltransferase and SUZ12 and EED structural components, leading to transcriptional repression through trimethylation of histone H3 at lysine 27 (H3K27me3) (20).

3-Deazaneplanocin A (DZNep) is the cyclopentanyl analog of 3-deazaadenosine that inhibits the activity of *S*-adenosylhomocysteine hydrolase, the enzyme responsible for reversible hydrolysis of *S*-adenosylhomocysteine to adenosine and homocysteine (21, 22), leading to the indirect inhibition of various *S*-adenosylmethionine-dependent methylation reactions (23). Thus, DZNep can be considered as an inhibitor of *S*-adenosylmethionine-dependent methytransferase. DZNep has recently been reported to decrease protein levels of PRC2 (24). Because DZNep can induce efficient apoptotic cell death in cancer cells but not in normal cells (24), it has been widely examined as a possible epigenetic therapeutic agent for the treatment of various cancers, including lung cancer (25), gastric cancer (26), myeloma (27), acute myeloid leukemia (28), and lymphoma (29). However, regardless of the evidence for the potential association of PRC2 with GATA-1 as described above, the impact of DZNep on erythropoiesis has remained unknown. In the present study, we examined the effects of DZNep in the K562 erythroid cell line and further extended the analysis to human primary erythroblasts.

## EXPERIMENTAL PROCEDURES

### 

#### 

##### Reagents

DZNep was purchased from Cayman Chemical Co. (Ann Arbor, MI) and was reconstituted with ethanol or DMSO (Sigma).

##### Cell Culture

Cells were grown in a humidified incubator at 37 °C with 5% CO_2_. The human K562 erythroleukemia cell line was maintained in RPMI 1640 medium containing 10% fetal bovine serum (Biowest, Miami, FL) and 1% penicillin-streptomycin (Sigma).

To differentiate erythroblasts from human CD34-positive cells derived from cord blood mononuclear cells, fresh cord blood (Riken BRC Cell Bank, Tsukuba, Japan) was overlaid on Ficoll-Paque PLUS (GE Healthcare). Samples were centrifuged at 1500 rpm for 30 min at room temperature. The upper layer was removed, mononuclear cells were washed twice with phosphate-buffered saline (PBS), and CD34-positive cells were then separated using a magnetic activated cell sorter system (Miltenyi Biotec, Auburn, CA). The CD34-positive cells were expanded with StemPro-34 SFM (Invitrogen) supplemented with stem cell factor (100 ng/ml), interleukin-3 (IL-3) (50 ng/ml), and granulocyte macrophage colony-stimulating factor (GM-CSF) (25 ng/ml) for 5 days (16, 30). Subsequently, the CD34-positive cells were differentiated into erythroid cells with StemSpan SFEM (StemCell Technologies, Vancouver, Canada) containing 100 ng/ml stem cell factor, 33.3 ng/ml Flt3-L, 13.3 ng/ml BMP4, 13.3 ng/ml IL-3, 2.67 units/ml erythropoietin, 1 μm hydrocortisone (Sigma) for up to 7 days, essentially as described previously (31). IL-3, stem cell factor, Flt3-L, BMP4, and GM-CSF were purchased from Peprotech (Rock Hill, NJ), and erythropoietin was a kind gift from Kyowa Hakko Kirin Co. Ltd. (Tokyo, Japan).

The use of cord blood samples for the study was approved by the ethics committee of Tohoku University. Ethical considerations according to the Declaration of Helsinki were followed.

##### Assay of Erythroid Differentiation with Benzidine Staining

Erythroid differentiation of K562 cells as well as primary erythroblasts was scored by benzidine staining as described previously (32). Benzidine (*o*-dianisidine) was obtained from Sigma. Benzidine staining-positive cells were quantified by light microscopy (*n* = 600). Viable cells were counted with trypan blue stain solution (Invitrogen).

##### Gene Transfer and Vectors

Overexpression of ETO2 was conducted with pBABE-puro vector as described previously (16). Briefly, the expression vector (10 μg) was transfected into K562 cells by Amaxa Cell Line Nucleofector II with the program T-016 (Amaxa Biosystems, Köln, Germany), according to the manufacturer's instructions.

##### Silencing of Gene Expression by siRNA

For siRNA-mediated transient human ETO2 and EZH2 knockdown in K562 cells, siGENOME siRNA SMART pool (Thermo Scientific Dharmacon, Lafayette, CO) was used. The antisense sequences of the siRNA for human *CBFA2T3* (which encodes ETO2 protein) were UGAAUGAGGUGAAGCGGCA, CAGCGGAGGUGAAGACGCA, CCAAAGAGAACGGGUCAGA, and CAUUGACGAUCGAGGAGUU, and for human *EZH2*, they were CAAAGAAUCUAGCAUCAUA, GAGGACGGCUUCCCAAUAA, GCUGAAGCCUCAAUGUUUA, and GAAUGGAAACAGCGAAGGA. As a negative control, siGENOME non-targeting siRNA pool 1 (Thermo Scientific Dharmacon) was used. siRNA was transfected into K562 cells using an Amaxa Cell Line Nucleofector II (Amaxa Biosystems) with the T-016 program as described previously (16). siRNA was transfected twice at 0 and 24 h, and the cells were harvested at 48 h.

##### Real-time Quantitative RT-PCR

Total RNA was purified with TRIzol (Invitrogen), and aliquots of 1 μg of purified total RNA were used to synthesize cDNA with ReverTra Ace qPCR RT Master Mix with gDNA Remover (Toyobo, Tokyo, Japan). Reaction mixtures (20 μl) for real-time quantitative RT-PCR consisted of 2 μl of cDNA, 10 μl of Quantitect SYBR Green PCR kit (Qiagen, Hilden, Germany), and appropriate primers. Primer sequences are available upon request. Product accumulation was monitored by measuring SYBR Green fluorescence and normalized relative to GAPDH or 28 S mRNA (10, 16, 30).

##### Microarray Analysis

For expression profiling to identify the DZNep-regulated and ETO2-regulated gene ensemble, SurePrint G3 Human GE 8×60K Microarrays (G4851B) were used according to the manufacturer's instructions (Agilent, Palo Alto, CA) (12, 16). Gene ontology analysis was conducted using the DAVID bioinformatics program. For expression profiling to identify the EZH2-regulated gene ensemble, a Human Oligo Chip 25k (Toray, Tokyo, Japan) was used essentially as described previously (30). For global normalization, the background value was subtracted and subsequently adjusted to the average signal value of 25.

##### Western Blotting Analysis

Whole-cell extracts were prepared by boiling cells for 10 min in SDS sample buffer (25 mm Tris, pH 6.8, 2% β-mercaptoethanol, 3% SDS, 0.1% bromphenol blue, 5% glycerol) at 1 × 10^7^ cells/ml. Extracts from 1–2 × 10^5^ cells were resolved by SDS-PAGE and transferred to Hybond-P (GE Healthcare). The proteins were measured semiquantitatively with ECL-Plus (GE Healthcare) and CL-X Posure^TM^ Film (Thermo Scientific). Each band was quantified with ImageJ software. For calculating relative intensity, control samples were set to 1.

##### Quantitative Chromatin Immunoprecipitation (ChIP) Analysis

Real-time PCR-based quantitative ChIP analysis was performed essentially as described (10, 16). Cells were cross-linked with 1% formaldehyde for 10 min at room temperature. The nuclear lysate was sonicated to reduce DNA fragment length using a Sonifier cell disruptor (Branson, Danbury, CT). The protein-DNA complexes were immunoprecipitated by specific antibody and Protein A- or Protein G-Sepharose (Sigma). Immunoprecipitated DNA fragments were quantified by real-time PCR to amplify regions of 75–150 bp overlapping with the appropriate motif. The product was measured by SYBR Green fluorescence in 20-μl reactions, and the amount of product was determined relative to a standard curve generated from titration of input chromatin. Analysis of postamplification dissociation curves indicated that primer pairs generated single products. Primer sequences are available upon request.

##### Antibodies

Antibodies to ETO2 (C-20), P27 (C-19), and actin (I-19) were obtained from Santa Cruz Biotechnology, Inc. Anti-EZH2 antibodies were obtained from Cell Signaling Technology (D2C9) (Danvers, MA) and Active Motif (Carlsbad, CA). Anti-α-tubulin antibody (CP06) and normal rabbit serum were obtained from Calbiochem. Anti-trimethyl-histone H3 (Lys-27) antibody (catalog no. 07-449) was obtained from Millipore (Temecula, CA). Anti-histone H3 (acetyl-Lys-9) antibody (ab4441), control IgG (rabbit), and H3 (ab1791) were obtained from Abcam (Cambridge, MA). Anti-HDAC antibodies were obtained from Cell Signaling Technology (catalog no. 9928). Phycoerythrin-labeled anti-glycophorin A (JC159) and anti-CD34 (BIRMA-K3) antibodies were obtained from Dako (Kyoto Japan).

##### Flow Cytometry

The cells collected from cultures were washed twice with PBS (Sigma). The cells were then incubated with relevant antibodies, washed twice with PBS, and analyzed using FACSCalibur or LSRFortessa^TM^ (BD Biosciences). The collected data were processed with FlowJo software.

##### Cell Cycle Analysis

The cells were washed twice with PBS and fixed in 70% ethanol at −20 °C. Fixed cells were subjected to centrifugation and then resuspended in 0.1% Triton X-100 (Sigma), 200 μg/ml RNase A (Invitrogen), and 20 μg/ml propidium iodide (Sigma) to label the DNA. DNA content was measured using LSRFortessa^TM^ (BD Biosciences).

##### Statistics

Statistical significance was assessed by two-sided Student's *t* test. To rank the gene ontology terms, corrected *p* values were calculated based on the Benjamini-Yakutieli method as described previously (33). In all analyses, *p* < 0.05 was taken to indicate statistical significance.

## RESULTS

### 

#### 

##### DZNep Treatment Depletes EZH2 Protein and Inhibits Cell Growth in K562 Cells

**FIGURE 1.**
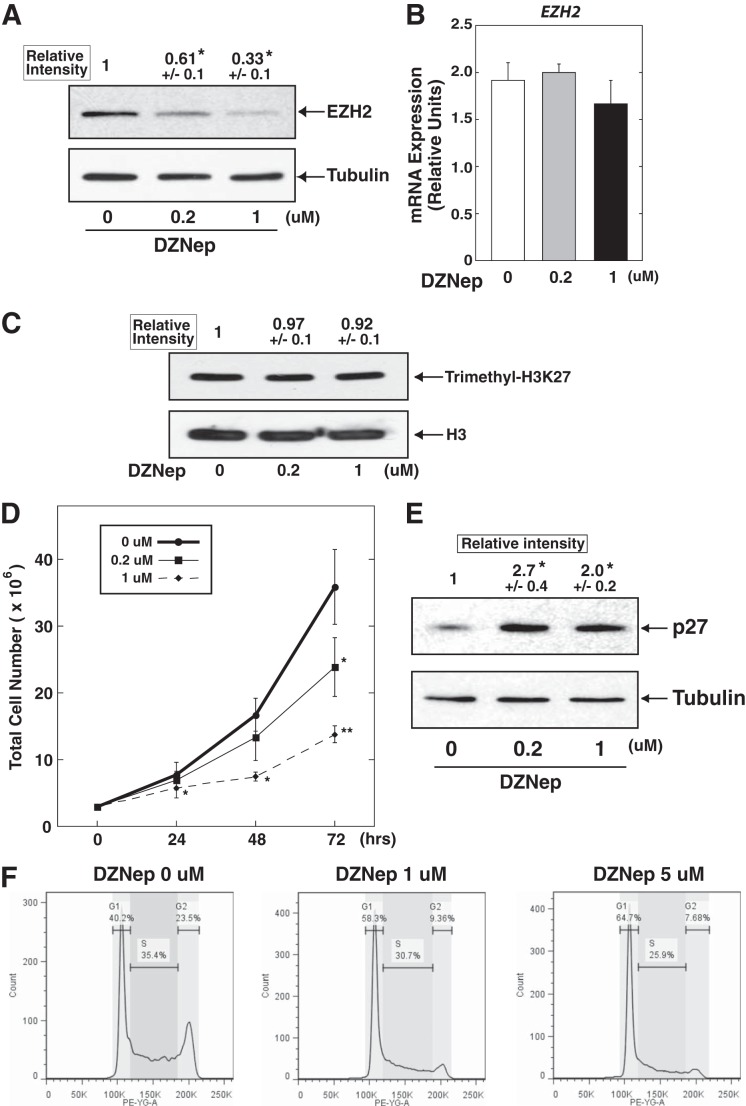
**DZNep treatment suppressed proliferative activity of K562 cells.** *A* and *B*, Western blotting to detect EZH2 protein (*A*) and mRNA (*B*) in DZNep-treated K562 cells. The K562 cells were treated with DZNep (0.2 and 1 μm, respectively) for 72 h. EZH2 protein signal was quantified and normalized to that of α-tubulin (*n* = 3, mean ± S.E. (*error bars*)). Statistical analyses were conducted by comparing with control signal (defined as 1). *, *p* < 0.05. mRNA expression was normalized relative to that of *GAPDH* (*n* = 4, mean ± S.E.). *C*, Western blotting to assess H3K27me3 level in DZNep-treated K562 cells. K3K27me3 level was quantified and normalized to that of H3 (*n* = 3, mean ± S.E.). Statistical analyses were conducted by comparing with control signal (defined as 1). *D*, changes in total cell number after DZNep treatment. Statistical significance was calculated by comparison with the control (0 μm) condition at each time point. *, *p* < 0.05; **, *p* < 0.01. *E*, Western blotting to detect p27 protein in DZNep-treated K562 cells. p27 protein signal was quantified and normalized to that of α-tubulin (*n* = 3, mean ± S.E.). Statistical analyses were conducted by comparing with control signal (defined as 1). *, *p* < 0.05. *F*, effect of DZNep (1 and 5 μm, respectively, for 24 h) on cell cycle in K562 cells. The percentage of cells in each cell cycle was measured using a flow cytometer. Representative data of three independent experiments are shown. In all analyses, we observed increased G_1_ as well as decreased G_2_ phase cells.

We first examined the effects of DZNep treatment based on K562 erythroleukemia cells. Cells were treated with DZNep at a concentration of 0.2 or 1 μm for 72 h, employing the dosages reported previously (25, 28). As shown in Fig. 1*A*, we confirmed the significant reduction of EZH2 protein levels, whereas *EZH2* mRNA expression was not significantly affected (Fig. 1*B*), suggesting that EZH2 level was regulated at the posttranscriptional level. Although DZNep treatment did not alter H3K27me3 levels (Fig. 1*C*), the treatment inhibited cell growth (Fig. 1*D*), accompanied by significant accumulation of the cell cycle regulator p27 (Fig. 1*E*) as well as G_1_ cell cycle arrest (Fig. 1*F*). These observations were similar to those of the previous study based on a lung cancer cell line (25). Intriguingly, we did not observe a significant increase in the frequency of apoptotic cells under identical conditions (data not shown). These results suggested that DZNep-mediated inhibition of cell growth is independent of its effect on H3K27me3.

##### DZNep Treatment Induced Erythroid Differentiation of K562 Cells

**FIGURE 2.**
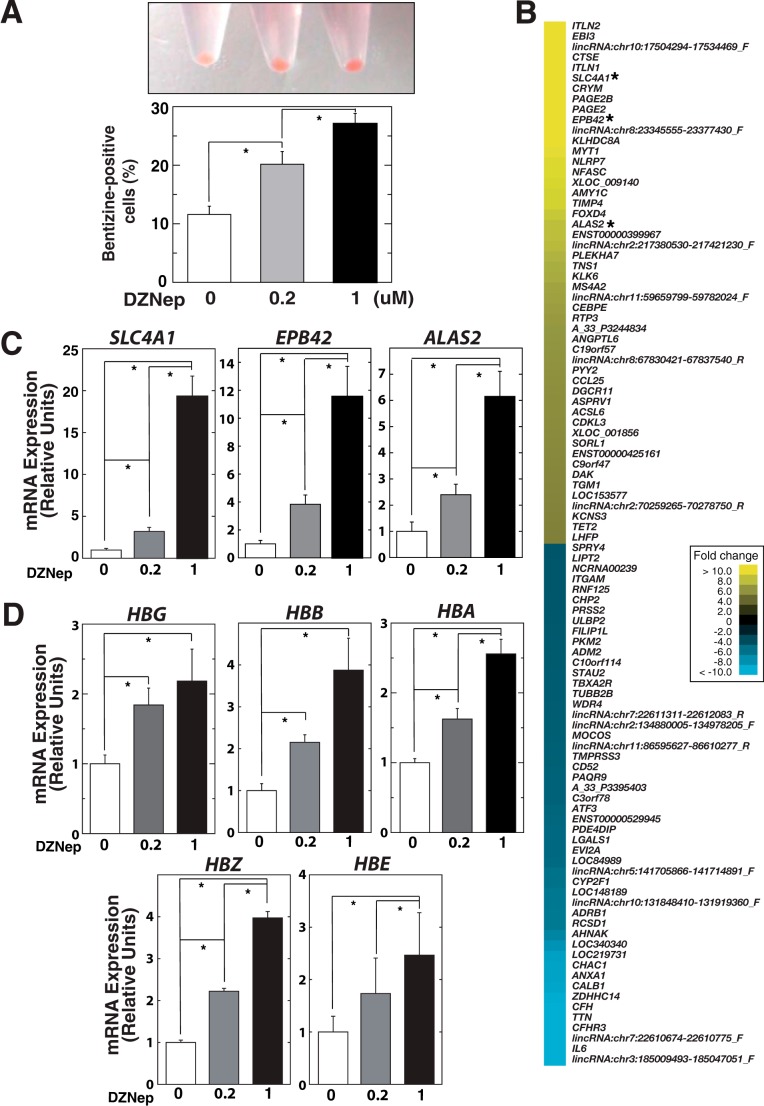
**DZNep treatment induces erythroid differentiation of K562 cells.** *A*, cell pellet (*top*) and the percentage of benzidine stain-positive cells (*bottom*) after DZNep treatment in K562 cells. The cells were treated with DZNep at doses of 0.2 and 1 μm for 72 h. *B*, expression profiling based on K562 cells treated with DZNep at a dose of 1 μm or control, using the SurePrint G3 Human GE 8×60K microarray (Agilent). The *heat map* depicts the -fold change resulting from DZNep treatment. The top 50 up-regulated and down-regulated genes are shown. *C* and *D*, quantitative RT-PCR-based validation analysis (*n* = 4, mean ± S.D. (*error bars*)). mRNA levels were normalized relative to *GAPDH*. *, *p* < 0.05.

Interestingly, we found that DZNep treatment induced hemoglobinization of K562 cells, as determined from the percentage of cells showing positive benzidine staining (Fig. 2*A*). We next conducted transcriptional profiling with untreated and DZNep-treated K562 cells (1 μm), demonstrating that 788 and 698 genes were up-regulated and down-regulated (>2-fold), respectively (supplemental Table 1). The DZNep-induced gene ensemble included prototypical erythroid genes, such as *SLC4A1*, *EPB42*, and *ALAS2* (Fig. 2*B*) (10), which were confirmed by quantitative RT-PCR analysis (Fig. 2*C*). Gene ontology analysis revealed significant enrichment of genes related to “oxygen transport” (*p* = 0.0062) in the DZNep-induced gene ensemble (Table 1). Quantitative RT-PCR analysis confirmed that DZNep treatment significantly increased globin gene expression (Fig. 2*D*). Taken together, these data suggested that DZNep induced the expression of representative erythroid genes and contributed to the erythroid differentiation of K562 cells.

**TABLE 1:** **Gene ontology analysis of DZNep-regulated genes** Genes showing >2-fold up-regulation and down-regulated genes induced by DZNep treatment (1 μm, 72 h) in K562 cells were analyzed with DAVID software.

Gene ontology term	*n*	*p* value
**Up-regulated**		
Oxygen transporter activity	4	0.0062
Transcription repressor activity	18	0.015
Guanyl-nucleotide exchange factor activity	11	0.016
3′,5′-Cyclic-nucleotide phosphodiesterase activity	4	0.034
*N*-Acyltransferase activity	7	0.039
Phosphoric diester hydrolase activity	7	0.041
Transition metal ion binding	99	0.048

**Down-regulated**		
Cofactor binding	16	0.0012
Small GTPase regulator activity	15	0.0079
Electron carrier activity	13	0.0085
Phosphatase activity	14	0.0086
Coenzyme binding	11	0.014
Protein dimerization activity	23	0.014
Oxidoreductase activity	4	0.018
Transcription corepressor activity	9	0.026
Lipid binding	19	0.029
Actin binding	15	0.030
FAD binding	6	0.031
Cysteine-type endopeptidase activity	6	0.031
Rho guanyl-nucleotide exchange factor activity	6	0.035
Transcription factor activity	34	0.037
Steroid dehydrogenase activity	4	0.039
Indanol dehydrogenase activity	2	0.048

##### EZH2 Inhibition Has No Significant Effect on Erythroid Gene Expression in K562 Cells

**FIGURE 3.**
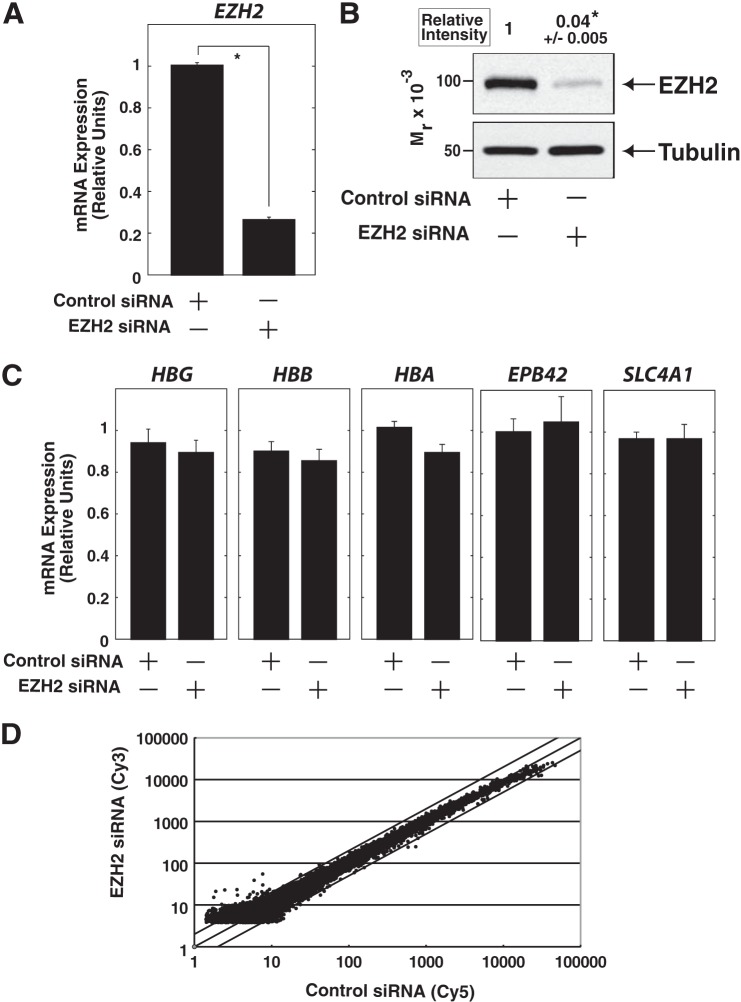
**Transient EZH2 knockdown has no significant effect on the expression of erythroid genes in K562 cells.** *A* and *B*, siRNA-mediated EZH2 knockdown in K562 cells. Quantitative RT-PCR analysis (*A*) and anti-EZH2 Western blotting of whole-cell extracts (*B*) are shown. EZH2 protein signal was quantified and normalized to that of α-tubulin (*n* = 3, mean ± S.E. (*error bars*)). Statistical analyses were conducted by comparing with control signal (defined as 1). *, *p* < 0.05. mRNA expression was normalized relative to that of *GAPDH* (*n* = 3, mean ± S.E.). *, *p* < 0.05. *C*, quantitative RT-PCR analysis for erythroid genes (analyzed in Fig. 2, *C* and *D*). mRNA levels were normalized relative to *GAPDH* (*n* = 3, mean ± S.E.). *D*, microarray profiling based on the Human Oligo Chip 25K (Toray). There was a high degree of similarity in the expression profile between control and EZH2 siRNA-treated K562 cells (*r* = 0.977).

To examine whether the observed results of DZNep treatment (Fig. 2) were due to the direct inhibition of EZH2 or to some hitherto unrecognized effects, we conducted siRNA-mediated knockdown of EZH2 in K562 cells (Fig. 3, *A* and *B*). Quantitative RT-PCR analysis demonstrated that EZH2 knockdown had no significant effect on the expression of erythroid lineage-related genes (Fig. 3*C*). Concomitantly, the knockdown did not exhibit significant changes in the percentages of benzidine staining-positive cells (data not shown). Furthermore, transcription profiles of the genes in the quantitative range of the array were similar between control and EZH2 siRNA-treated K562 cells (*r* = 0.977) (Fig. 3*D*), except for obvious down-regulation of *EZH2* (2.96-fold). Taken together with our observation that DZNep treatment did not affect global H3K27me3 level in K562 cells (Fig. 1*C*), we consider that DZNep-mediated erythroid differentiation may not be directly related to EZH2 inhibition.

##### DZNep Reduced Protein Level of the Hematopoietic Corepressor, ETO2, in K562 Cells

**FIGURE 4.**
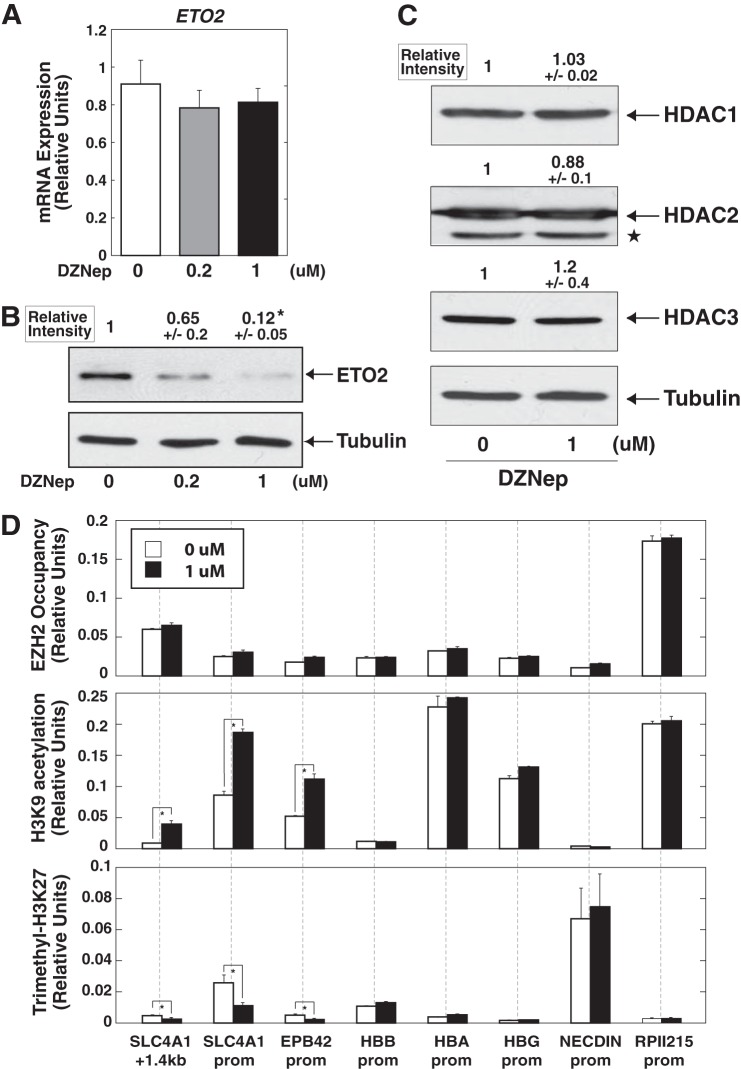
**ETO2 protein level decreased with DZNep treatment in K562 cells.** *A* and *B*, quantitative RT-PCR analysis (*A*) and anti-ETO2 Western blotting (*B*) of DZNep-treated K562 cells. K562 cells were treated with DZNep (0.2 and 1 μm, respectively) for 72 h. ETO2 protein signal was quantified and normalized to that of α-tubulin (*n* = 3, mean ± S.E. (*error bars*)). Statistical analyses were conducted by comparing with control signal (defined by 1). *, *p* < 0.05. mRNA expression was normalized relative to that of *GAPDH* (*n* = 4, mean ± S.E.). *, *p* < 0.05. *C*, Western blotting to detect class I HDACs in DZNep-treated K562 cells. The K562 cells were treated with 1 μm DZNep for 72 h. Protein signals for each HDAC were quantified and normalized to that of α-tubulin (*n* = 3, mean ± S.E.). Statistical analyses were conducted by comparing with control signal (defined as 1). α-Tubulin was used as a loading control. *Star*, cross-reactive band. *D*, quantitative ChIP analysis to examine EZH2 occupancy and the levels of H3K9ac and H3K27me3 at various loci (mean ± S.D., *n* = 3) in DZNep-treated K562 cells. *NECDIN* and *RPII215* promoters were included as inactive and active promoter regions, respectively. Mean preimmune (or IgG control) signal did not exceed 0.01. *, *p* < 0.05.

The molecular mechanisms by which DZNep induced erythroid differentiation remain unclear. However, the preferential up-regulation of *SLC4A1* and *EPB42* among prototypical erythroid-related genes (Fig. 2, *B* and *C*) suggests that transcriptional corepressor ETO2 may play an important role, because we demonstrated previously that *Slc4a1* and *Epb4.2* were highly sensitive to ETO2 knockdown in murine erythroid cells (G1E-ER-GATA-1 cells (34)) (12). Therefore, we examined ETO2 expression in DZNep-treated K562 cells. The results indicated a significant reduction of ETO2 protein level, whereas *ETO2* mRNA expression was not significantly affected (Fig. 4, *A* and *B*), implying the involvement of posttranscriptional regulation mediated by DZNep. We also confirmed that the expression of class I HDACs, which interacts with ETO2 (13), was not significantly compromised by DZNep treatment (Fig. 4*C*).

Quantitative ChIP analysis demonstrated that EZH2 occupancy at promoters of DZNep-induced erythroid-related genes was unchanged by DZNep treatment (Fig. 4*D*). On the other hand, the treatment significantly increased acetylation of histone H3 at lysine 9 (H3K9ac) at the *SLC4A1* and *EPB42* loci without influencing this marker at globin as well as control promoters, including inactive and active *NECDIN* and *RPII215* promoters, respectively (Fig. 4*D*). Interestingly, H3K27me3 levels were also significantly decreased at the *SLC4A1* and *EPB42* loci (Fig. 4*D*).

**FIGURE 5.**
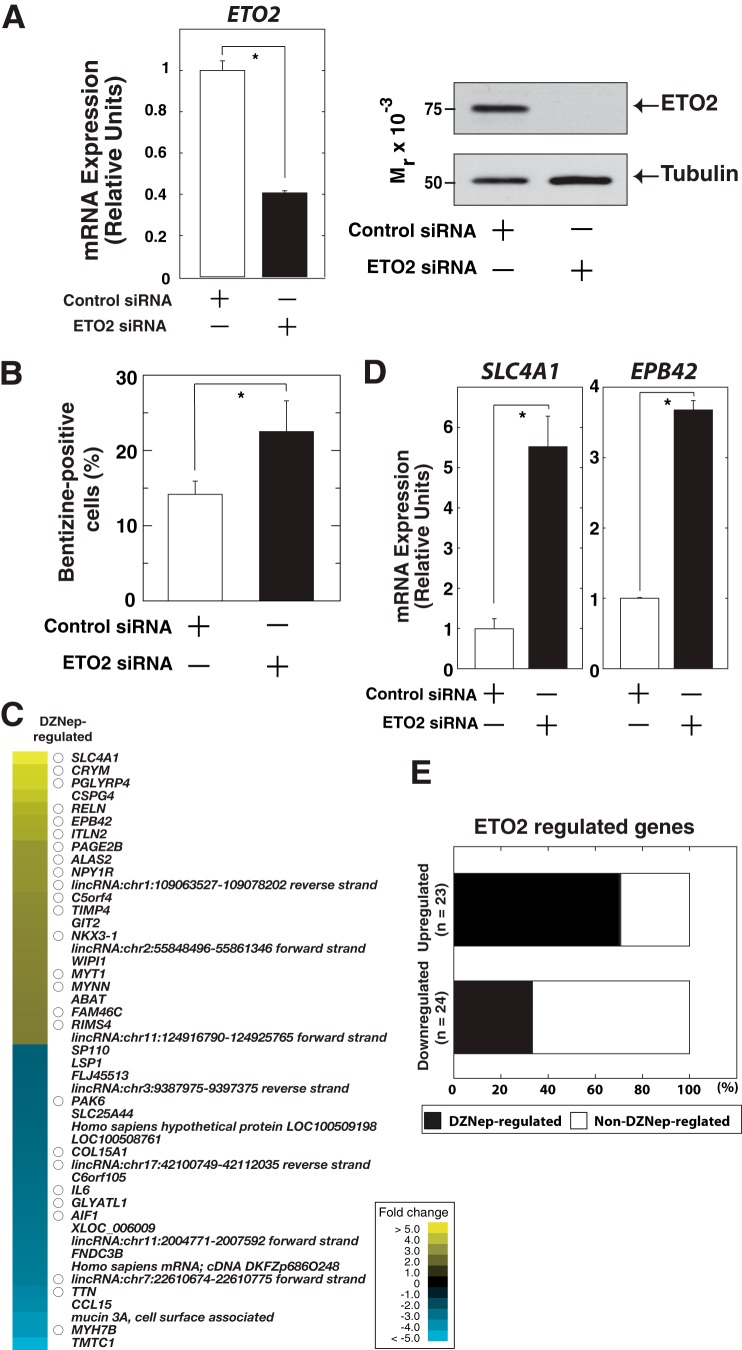
**Overlap between ETO2-regulated and DZNep-regulated gene ensemble.** *A*, siRNA-mediated ETO2 knockdown in K562 cells. The siRNA was transfected twice at 0 and 24 h, and the cells were harvested at 48 h. Quantitative RT-PCR analysis (*left*) and anti-ETO2 Western blotting of whole-cell extracts (*right*) are shown. α-Tubulin was used as a loading control. mRNA expression was normalized relative to that of *GAPDH* (*n* = 3, mean ± S.E. (*error bars*)). *, *p* < 0.05. *B*, the percentage of benzidine stain-positive cells after ETO2 knockdown in K562 cells (*n* = 3, mean ± S.E.). *, *p* < 0.05. *C*, expression profiling in K562 cells treated with control or anti-ETO2 siRNA, using the SurePrint G3 Human GE 8×60K microarray (Agilent). The heat map depicts the -fold change resulting from ETO2 knockdown. The genes showing >3-fold changes are shown. The overlap with DZNep-regulated genes (>2-fold changes; Fig. 2) is indicated by a *circle. D*, quantitative RT-PCR-based validation analysis for *SLC4A1* and *EPB42*. mRNA levels were normalized relative to *GAPDH* (*n* = 3, mean ± S.E.). *, *p* < 0.05. *E*, percentage of DZNep-regulated genes among the ETO2 target gene ensemble (up-regulated, *n* = 23; down-regulated, *n* = 24).

**FIGURE 6.**
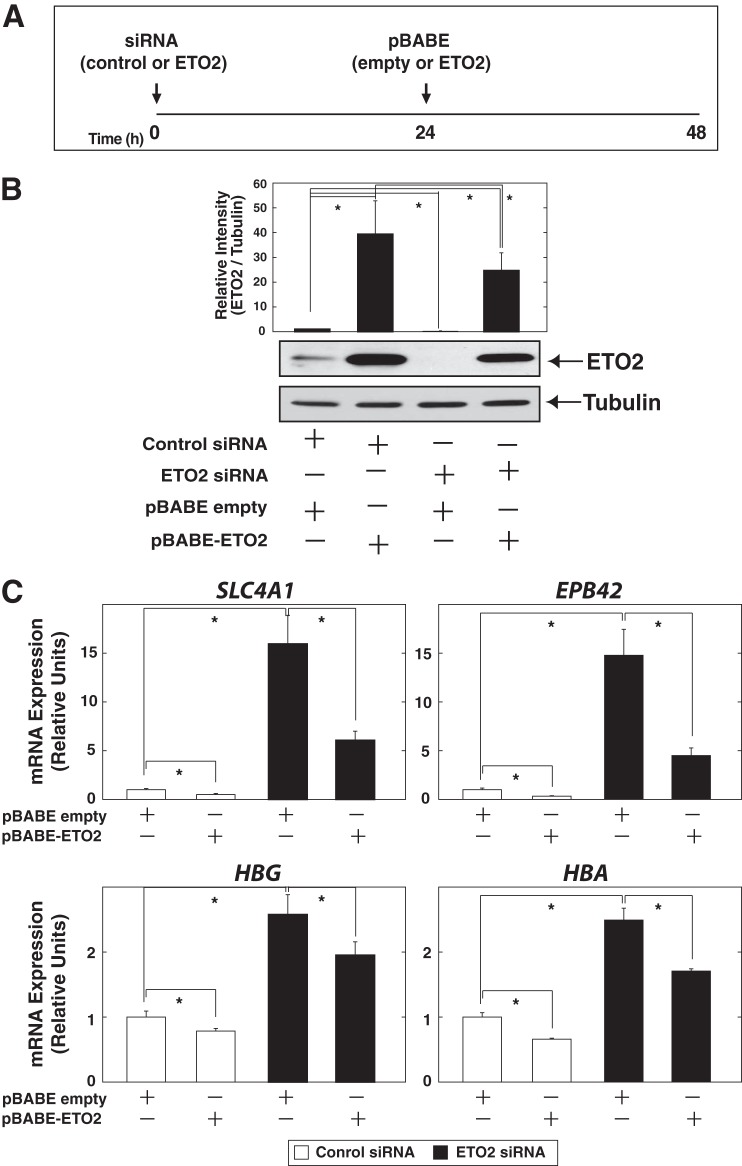
**Erythroid-related gene expression was induced by siRNA-mediated knockdown of ETO2 in K562 cells.** *A*, experimental strategy for ETO2 overexpression after siRNA-mediated ETO2 knockdown in K562 cells. *B*, Western blotting analysis of whole-cell extracts from each condition. Anti-ETO2 antibody was used to detect endogenous as well as exogenous ETO2 protein. ETO2 protein signal was quantified and normalized to that of α-tubulin (*n* = 3, mean ± S.E. (*error bars*)). Statistical analyses were conducted by comparing with control signal (defined as 1). *, *p* < 0.05. *C*, quantitative RT-PCR analysis of *SLC4A1*, *EPB42*, *HBG*, and *HBA* expression in ETO2-overexpressed K562 cells, transfected with anti-ETO2 or control siRNA. The expression level of each target gene relative to that of *GAPDH* was calculated (*n* = 3, mean ± S.E.; *, *p* < 0.05).

**FIGURE 7.**
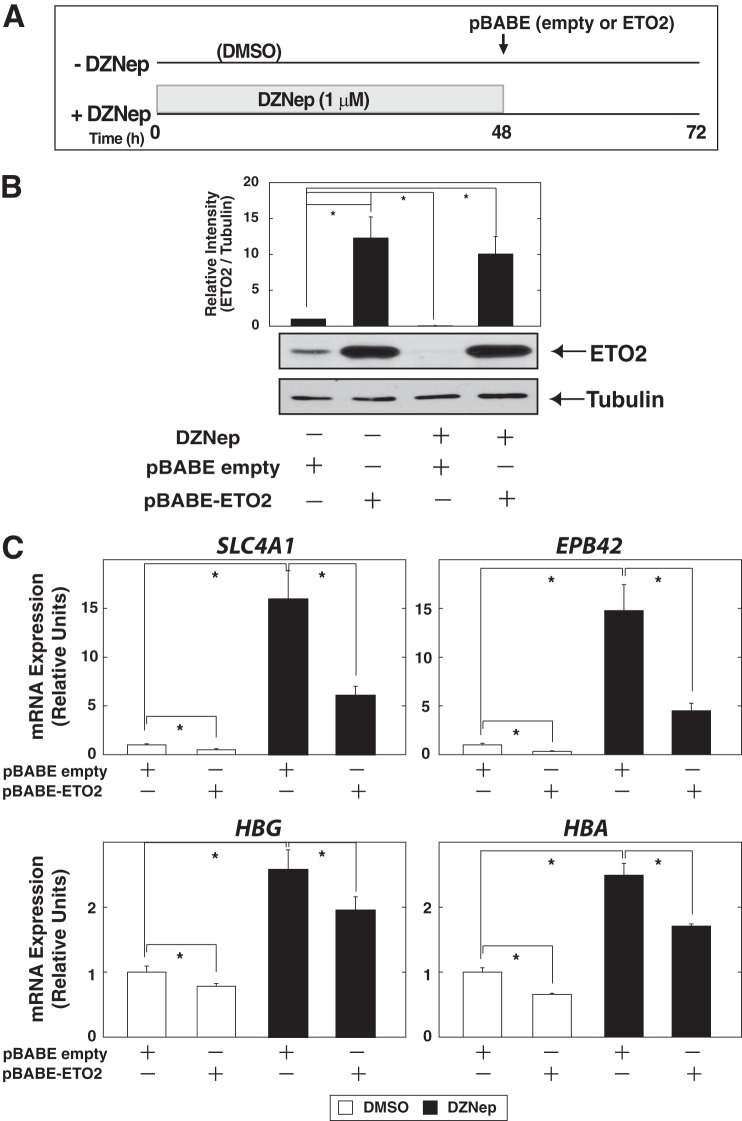
**ETO2 contributes to DZNep-mediated erythroid differentiation in K562 cells.** *A*, experimental strategy for ETO2 overexpression after DZNep treatment in K562 cells. *B*, Western blotting analysis of whole-cell extracts from ETO2-overexpressed K562 cells, treated with DZNep or DMSO. Anti-ETO2 antibody was used to detect endogenous as well as exogenous ETO2 protein. ETO2 protein signal was quantified and normalized to that of α-tubulin (*n* = 3, mean ± S.E. (*error bars*)). Statistical analyses were conducted by comparing with control signal (defined as 1). *, *p* < 0.05. *C*, quantitative RT-PCR analysis of *SLC4A1*, *EPB42*, *HBG*, and *HBA* expression in ETO2-overexpressed K562 cells, treated with DZNep or DMSO. The expression level of each target gene relative to that of *GAPDH* was calculated (*n* = 3, mean ± S.E.; *, *p* < 0.05).

To determine the contribution of the decrease in ETO2 due to DZNep treatment to the total DZNep-regulated gene ensemble, we conducted expression profiling to identify ETO2-regulated genes, which were subsequently compared with the DZNep-regulated gene ensemble (Fig. 2). ETO2 was transiently knocked down in K562 cells with anti-ETO2 siRNA. As shown in Fig. 5*A*, we confirmed significant decreases in ETO2 mRNA and protein levels. In addition, the percentage of benzidine staining-positive cells and expressions of globin genes were significantly increased after ETO2 knockdown (Figs. 5*B* and 6). Transcriptional profiling of K562 cells with control and anti-ETO2 siRNA demonstrated that 94 and 73 genes were up-regulated and down-regulated (>2-fold), respectively (supplemental Table 2). We confirmed that *SLC4A1* and *EPB42* were strongly up-regulated after ETO2 knockdown (Fig. 5, *C* and *D*), which were repressed again by overexpression of ETO2 (Fig. 6), suggesting the specificity of ETO2 knockdown. Interestingly, 69.5 and 37.5% of ETO2 knockdown-up-regulated and -down-regulated genes, respectively, were also regulated by DZNep treatment (Fig. 5*E* and supplemental Table 2). To further address if DZNep-mediated ETO2 decrease is truly responsible for erythroid differentiation of K562 cells, we rescued ETO2 expression after DZNep treatment (Fig. 7, *A* and *B*). As shown in Fig. 7*C*, we confirmed that the expressions of erythroid-related genes were induced by DZNep treatment, which was inhibited by ETO2 overexpression. Therefore, we consider that ETO2 may be at least one of the important factors contributing to the processes.

##### DZNep Induces Erythroid Gene Expression in CD34-positive Cell-derived Primary Erythroblasts

**FIGURE 8.**
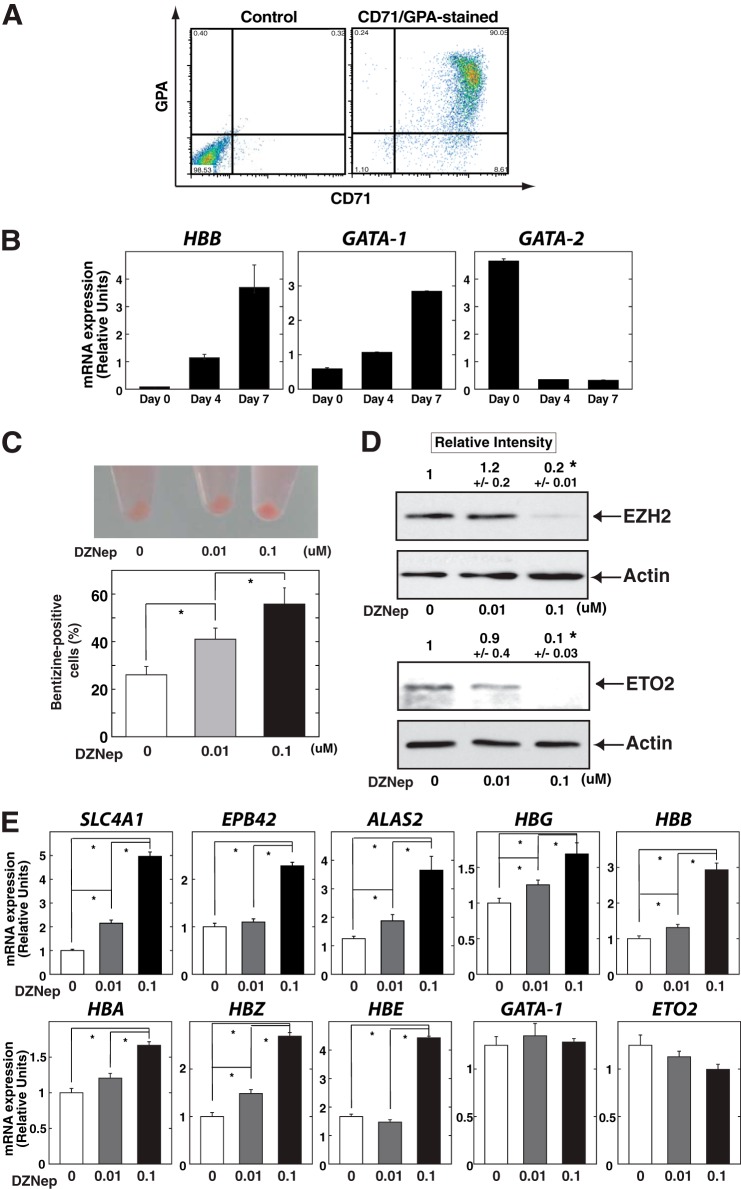
**DZNep induces erythroid differentiation in primary erythroblasts.** *A*, CD34-positive cells were cultured in erythroid medium, and the expressions of glycophorin A and CD71 were examined on day 7 using FACSCalibur (BD Biosciences). *B*, quantitative RT-PCR analysis for *HBB*, *GATA-1*, and *GATA-2* transcripts on days 0, 4, and 7 of culture. Product accumulation was normalized relative to 28 S mRNA (mean ± S.E. (*error bars*), *n* = 3). *C*, cell pellet (*top*) and the percentage of cells showing positive benzidine staining (*bottom*) after DZNep treatment in primary erythroblasts. The cells were treated with DZNep at doses of 0.01 and 0.1 μm with erythroid differentiation medium for 96 h. *D*, Western blotting to detect EZH2 (*top*) and ETO2 (*bottom*) in primary erythroblasts. Protein signals for EZH2 and ETO2 were quantified and normalized to that of actin (*n* = 3, mean ± S.E.). Statistical analyses were conducted by comparing with control signal (defined by 1). *, *p* < 0.05. Actin was used as a loading control. *E*, quantitative RT-PCR analysis after DZNep treatment in human primary erythroblasts. The cells were treated with DZNep at doses of 0.01 and 0.1 μm for 96 h. Product accumulation was normalized relative to 28 S mRNA (mean ± S.E., *n* = 3). *, *p* < 0.05.

We analyzed the effects of DZNep in the K562 erythroleukemia cell line, but the phenotypic and karyotypic differences form normal erythroblasts are difficult to interpret. Therefore, we moved to an *ex vivo* model of human erythroid differentiation, starting from cord blood-derived CD34-positive cells. In the present study, isolated CD34-positive cells were first expanded in serum-free medium containing stem cell factor, IL-3, and GM-CSF for 5 days and subsequently underwent erythroid differentiation based on serum-free medium as described previously (16, 31). Erythroid differentiation was evaluated by measuring the proportion of cell surface CD71- and glycophorin A-positive cells by flow cytometry analysis (Fig. 8*A*). We performed quantitative RT-PCR analysis to analyze the expression pattern of genes involved in the erythroid pathway. As shown in Fig. 7*B*, erythroid-related genes, such as the β-globin gene (*HBB*), were strongly induced along with differentiation. It has been demonstrated that, prior to the increase in GATA-1 expression during erythroid differentiation, GATA-2 is expressed in hematopoietic stem cells (4), and GATA-1 directly represses *GATA-2* transcription at the *GATA-2* locus (termed the “GATA switch”) (1). Likewise, we confirmed the down-regulation of *GATA-2* and subsequent increase of GATA-1 in our analysis (Fig. 8*B*).

Based on the primary erythroid differentiation system, cells were treated with DZNep at 0.01 and 0.1 μm for 96 h. Similar to the analysis based on K562 cells (Fig. 2), the cell pellet appeared reddish in color and showed a significantly higher percentage of benzidine-positive cells (Fig. 8*C*). Western blotting analysis confirmed the significant decrease of EZH2 as well as ETO2 protein level at 0.1 μm (Fig. 8*D*). Furthermore, quantitative RT-PCR analysis confirmed up-regulation of erythroid genes, including *SLC4A1*, *EPB42*, *ALAS2*, *HBG*, *HBB*, *HBA*, *HBZ*, and *HBE*, whereas GATA-1 and ETO2 were unaffected (Fig. 8*E*). Taken together, these data suggested that DZNep has the capacity to induce differentiation of primary erythroblasts, and the decrease of ETO2 may be partly involved in this process.

## DISCUSSION

Epigenetic drugs have been tested in preclinical and clinical cancer models. DNA methyltransferase inhibitors and histone deacetylase inhibitors have been approved for the treatment of some hematological malignancies (35). On the other hand, an inhibitor of *S*-adenosylmethionine-dependent methytransferase, DZNep, showed promising results against lung cancer (25), gastric cancer (26), myeloma (27), acute myeloid leukemia (28), and lymphoma (29). Furthermore, DZNep has been reported to modulate allogeneic T cell responses and may represent a novel therapeutic approach for treatment of graft *versus* host disease (36). Despite these promising results, there has been little focus on the effects of DZNep on normal hematopoiesis, especially erythropoiesis.

Although we confirmed that DZNep treatment reduced EZH2 protein level in K562 cells, the treatment did not affect H3K27me3 level (Fig. 1, *A–C*). Similar unexpected results were observed in myeloma as well as lung cancer cell lines (25, 27). These observations suggest that proteins other than EZH2 may be responsible for changes in H3K27me3. Alternatively, DZNep may also affect other histone methyltransferases that are not responsible for H3K27me3, such as those responsible for histone H3 Lys-9 trimethylation, histone H3 Lys-4 trimethylation, and histone H3 Lys-20 trimethylation (37), which may have an influence on K562 cells.

One of the most important findings of the present study was that DZNep treatment promotes erythroid differentiation (Figs. 2 and 8). Unexpectedly, siRNA-mediated direct inhibition of EZH2 had little influence on erythroid differentiation of K562 cells (Fig. 3), suggesting the dispensable role of EZH2. Consistent with this suggestion, a previous study in *Ezh2* conditional knock-out mice demonstrated that depletion of *Ezh2* in adult bone marrow did not significantly affect erythropoiesis (38). On the other hand, another report suggested that EZH2 is required for the transient repression of *Hes1*, a downstream target of the Notch signaling pathway, in murine erythroid cells (39), which supported a positive effect of EZH2 on erythroid differentiation. However, we did not observe the activation of *HES1* after EZH2 knockdown (Fig. 3*D*), suggesting that *HES1* may not be solely regulated by EZH2 in K562 cells. Taken together, these observations suggest that DZNep promotes erythroid differentiation of K562 cells, presumably through a mechanism that is not directly related to EZH2 inhibition.

Although the molecular mechanisms by which DZNep treatment promotes erythroid differentiation have remained elusive, we speculated that the decrease in hematopoietic corepressor ETO2 level might promote erythroid differentiation in K562 cells as well as primary erythroblasts (Figs. 4*B* and 8*D*). Our hypothesis is supported by the obvious overlap between the DZNep-activated and ETO2-repressed gene ensemble (Fig. 5, *C–E*), including preferential up-regulation of *SLC4A1* and *EPB42*, reported as ETO2-sensitive genes (10, 12), by DZNep treatment (Figs. 2*B* and 5*D*). Interestingly, DZNep treatment significantly increased H3K9ac but decreased H3K27me3 at the *SLC4A1* and *EPB42* loci (Fig. 4*D*), although DZNep treatment did not affect global H3K27me3 level (Fig. 1*C*). Unresponsiveness of these histone marks at globin gene promoters (Fig. 4*C*) might possibly be explained by the relatively weak induction of mRNA levels in our experimental conditions. Nevertheless, the findings were quite similar to those of previous reports based on ETO2 knockdown in G1E-ER-GATA-1 cells, indicating that H3K27me3 could be affected at ETO2-sensitive gene loci (12). Given its binding properties with HDACs (13), the increase in H3K9ac is consistent with this mechanism. On the other hand, the molecular mechanism by which ETO2 confers changes in H3K27me3 levels at the target gene loci remains unknown. Nevertheless, the present data based on K562 cells support the possibility that it may be a universal or physiological phenomenon that ETO2 regulates H3K27me3 levels at these loci, and further investigations are warranted to determine the underlying molecular mechanisms.

To our knowledge, there is no direct evidence supporting our hypothesis that DZNep decreases ETO2 protein level in erythroid cells. The decrease of ETO2 protein, with no effect on its mRNA level, suggests the possible involvement of posttranscriptional regulation by DZNep (Fig. 4, *A* and *B*). Because DZNep has been reported to induce ubiquitin ligases, which contribute to inhibition of EZH2 protein in a proteasome-dependent manner (24, 40), these ubiquitin ligases may have an impact on ETO2 protein level. Alternatively, given the RNA binding properties of ETO2 (41), DZNep-regulated RNA components may affect protein stability of ETO2.

An important implication of this study is that DZNep may be a new differentiation-inducing compound (42, 43), because the treatment significantly induced various globin genes, including fetal hemoglobin, in K562 cells (Fig. 2) as well as primary erythroblasts (Fig. 8). Known inducers of erythroid differentiation of K562 cells, such as hydroxyurea and 5-azacytidine, are also capable of inducing fetal globin induction when administered to normal erythroid cells alone or in combination (43), and these inducers have already been applied in clinical settings. Alternatively, DZNep may be applicable in “differentiation therapy” to treat various hematological and perhaps non-hematological malignancies, as represented by all-*trans*-retinoic acid for acute promyelocytic leukemia (44). It has already been demonstrated that DZNep treatment induces the differentiation of acute myeloid leukemia cell lines (HL-60 and OCI-AML3) (21, 28). Further experiments, including *in vivo* studies in experimental animals, are still necessary to conclusively determine the efficacy of DZNep. Furthermore, because DZNep may alter the expression of many genes that are essential for normal hematopoiesis, determining the precise mechanism underlying the promotion of erythroid differentiation by DZNep may lead to the development of more specific and effective drugs.

In conclusion, we have demonstrated that DZNep promotes erythroid differentiation. Our findings highlight the therapeutic potential of DZNep as a differentiation-inducing drug.

## Supplementary Material

Supplemental Data

## Footnotes

2 The abbreviations used are:

HDAC: histone deacetylase
H3K27m3: trimethylation of histone H3 at lysine 27
H3K9ac: acetylation of histone H3 at lysine 9
DZNep: 3-deazaneplanocin A.
