# Supplementary material for: 3-Deazaneplanocin A (DZNep), an Inhibitor of S-Adenosylmethionine-dependent Methyltransferase, Promotes Erythroid Differentiation
Source: J Biol Chem. 2014 Feb 3;289(12):8121–34. doi: 10.1074/jbc.M114.548651 (PMC3961643; doi:10.1074/jbc.M114.548651)
Supplement: Supplemental Data [file supp_289_12_8121__index.html]

3-Deazaneplanocin A (DZNep), an inhibitor of S-adenosyl-methionine-dependent methyltransferase, promotes erythroid differentiation — 3-Deazaneplanocin A (DZNep), an Inhibitor of S-Adenosylmethionine-dependent Methyltransferase, Promotes Erythroid Differentiation — DZNep Promotes Erythroid Differentiation — Supplemental Data 

# 3-Deazaneplanocin A (DZNep), an Inhibitor of *S*-Adenosylmethionine-dependent Methyltransferase, Promotes Erythroid Differentiation

## Supplemental Data

**Files in this Data Supplement:**

- Supplementary Table 1 (.xlsx, 273 KB) - Expression profiling of DZNep-treated K562 cells
- Supplementary Table 2 (.xlsx, 76 KB) - Expression profiling of ETO2-knockdowned K562 cells
